# Depressive and anxiety disorders among women with obesity

**DOI:** 10.1192/j.eurpsy.2021.909

**Published:** 2021-08-13

**Authors:** N. Kornetov, O. Yarosh, E. Sitnikova

**Affiliations:** 1 Department Of Psychiatry, Addiction And Psychotherapy, Siberian States Medical Unifersity, Tomsk, Russian Federation; 2 Psychiatry, Addiction, Psychotherapy, Siberian States Medical Unifersity, Tomsk, Russian Federation; 3 4th Year Student Of The Medical Faculty, Medical Unifersity, Tomsk, Russian Federation

**Keywords:** Depressive Disorder, Anxiety, Obesity, Body mass index, Depressive Disorder, Body Mass Index, Anxiety

## Abstract

**Introduction:**

Abdominal obesity is currently a growing problem in public health and has a high comorbidity with depressive and anxiety disorders. Obesity significantly decreases life quality, causes disability and decreases life expectancy.

**Objectives:**

The objective of this study was to examine anxiety and depressive symptoms among women, who received individual or group psychotherapy sessions due to obesity control.

**Methods:**

577 women aging from 18 to 65 were examined. Height and weight were measured, Body mass index (BDI) was calculated based on received data. Depressive symptoms were determined with the PHQ-9 questionnaire [Kroenke K, Spitzer RL, Williams JB]. Anxiety symptoms were determined with the GAD-7 questionnaire. The level of social adaptation was examined.

**Results:**

The prevalence of mild depression in our sample of women with obesity was 31.5%, 19.1% - moderate depression, 1% - severe depression, 48.4% had no depression symptoms. Anxiety symptoms were found in 38.2% of examined women, 61.8% showed no anxiety symptoms. Furthermore, when patients were divided into subgroups accordingly to BMI, anxiety was mostly registered among ones with normal BMI. An average correlation between indicators of anxiety and depressive symptoms was identified (r=0,62, p<0,05). Average correlation between indicators of anxiety and depression and the level of social adaptation (r=0,59 and r=0,48 relatively, p<0,05). Anxiety and depressive symptoms’ dependency on BMI was not established.

**Conclusions:**

The received data showed that anxiety and depression have high prevalence among women with obesity. The study will help medical specialists draw attention to high comorbidity between abdominal obesity and anxiety-depressive disorders.

